# Expression of hedgehog signal pathway in articular cartilage is associated with the severity of cartilage damage in rats with adjuvant-induced arthritis

**DOI:** 10.1186/s12950-015-0072-5

**Published:** 2015-03-28

**Authors:** Rong Li, Li Cai, Cheng-mu Hu, Ting-ni Wu, Jun Li

**Affiliations:** School of Pharmacy, Anhui Medical University, 81 Meishan Road, Hefei, 230032 Anhui China; Key Laboratory for Bioactivity of Natural Medicine of Anhui Province, Hefei, 230032 Anhui China; Department of Pathology, School of Basic Medicine, Anhui Medical University, 81 Meishan Road, Hefei, 230032 Anhui China

**Keywords:** Adjuvant-induced arthritis, Articular cartilage, Hedgehog signal pathway, Inflammation, Rheumatoid arthritis

## Abstract

**Background:**

Cartilage damage is a crucial step in rheumatoid arthritis (RA) disease progress while its molecular mechanisms are not fully understood. Here we investigated the expression of hedgehog (Hh) signal pathway in articular cartilage of adjuvant-induced arthritis (AIA) rats and its possible pathological role in cartilage damage.

**Methods:**

30 rats were divided into sham and AIA group (n = 15). Complete Freund’s adjuvant was used to induce AIA. Secondary paw swelling was measured on day 10, 14, 18, 22 and 26 after induction. Rats were sacrificed on day 26 and knee joints and cartilage tissues were collected. Paw swelling, cartilage histopathologic changes and OARSI scores were used to evaluate AIA in rats. The protein expression of Hh signal related genes (Shh, Ptch1, Smo and Gli1) in cartilage were assayed by immunohistochemistry. The mRNA levels of Shh, Ptch1, Smo, Gli1, type-II collagen (COII) and aggrecan in cartilage were assayed by real-time PCR. *In vitro* study, cultured AIA chondrocytes were treated with cyclopamine (a specific inhibitor of Hh signal) and the mRNA levels of Hh signal and ECM components (COII and aggrecan) were measured by real-time PCR.

**Results:**

Immunohistochemical results revealed that Shh, Ptch1, Smo and Gli1 proteins showed higher expression in the articular cartilage of AIA rats than those of sham rats. Real-time PCR results confirmed that Shh, Ptch1, Smo and Gli1 mRNA levels in cartilage tissues of AIA rats were significantly increased compared with those of sham rats (1.6, 1.4, 1.6, 2.0 fold, respectively). The mRNA levels of Shh, Ptch1, Smo, and Gli1 were associated with the severity of cartilage damage (indicated by OARSI scores, COII and aggrecan mRNA levels in cartilage). *In vitro*, cyclopamine effectively decreased the mRNA levels of Shh, Ptch1, Smo and Gli1, and increased the mRNA levels of COII and aggrecan in AIA chondrocytes, suggesting Hh signal inhibition might directly promote ECM production.

**Conclusions:**

Our findings present certain experimental evidence that Hh signal pathway is involved in the pathogenesis of cartilage damage in RA.

## Background

Rheumatoid arthritis (RA) is a chronic inflammatory disease that often leads to serious disability resulting from cartilage and joint destruction [1,2]. Articular cartilage damage is related to irreversible physical disability of RA and especial attention should be taken to therapeutic interference with cartilage damage [3]. However, most studies on the pathogenetic mechanisms of RA focused on synovial hyperplasia and inflammatory cell infiltration, the pathogenesis of cartilage damage has not received much attention. In physiological condition, cartilage homeostasis is maintained by a balance between synthesis and degradation of extracellular matrix (ECM) that is composed of fibrils containing type-II collagen (COII) and proteoglycans [4]. In RA, many pathological factors including proinflammatory cytokines, aggrecanases, matrix metalloproteinases and nitric oxide result in the imbalance between anabolic and catabolic processes of ECM, finally induce cartilage destruction [5]. Yet, the detailed molecular mechanisms underlying cartilage damage in RA are not fully understood.

Hedgehog (Hh) signal pathway regulates chondrocyte growth and differentiation during development and after birth [6,7]. Hh protein family consists of Indian hedgehog, Desert hedgehog and Sonic hedgehog (Shh, the best studied Hh protein with broadest expression pattern) [8]. Extracellular Hh protein binds to patched homologue 1 (Ptch1) and releases the inhibition of Ptch1 on Smoothened (Smo). Signaling by Smo results in the activation of transcription factors encoded by glioma-associated oncogene homolog (Gli) and consequent induction of Hh target genes (Gli1, Ptch1), and the levels of these transcripts are regarded as surrogate markers of Hh pathway activity [9,10].

Although the etiologies and pathologies of RA and osteoarthritis (OA) are different, the progressive cartilage damage is a marked feature of both diseases. The pathological role of Hh signal in cartilage damage has been demonstrated in OA disease. Hh signal was apparently activated in osteoarthritic cartilage and its blockage could be used as a therapeutic approach to inhibit cartilage degeneration in OA [11]. Hh signal expression correlated with OA progression and changes in chondrocyte morphology and gene expression was consistent with cartilage degradation in OA cartilage [12]. Interestingly, a recent study revealed that Shh signal pathway was activated in synovium of RA patients and in cultured fibroblast-like synoviocytes from RA patients *in vitro*, suggesting shh signal as a novel therapeutic target in RA [13]. However, there is no direct available study observing the expression of Hh signal in articular cartilage and its possible role in cartilage damage of RA. It is unknown whether the similar pathological mechanisms of Hh signal in OA apply to the cartilage damage in RA.

In the present study, we investigated the expression of Hh signal related genes (Shh, Ptch1, Smo and Gli1) at protein and mRNA levels in the articular cartilage of rats with adjuvant-induced arthritis (AIA). Then we correlated mRNA levels of Shh, Ptch1, Smo and Gli1 with the severity of cartilage damage in AIA rats. *In vitro*, we observed the potential effect of cyclopamine (a specific inhibitor of Smo) on mRNA expression of Hh signal and ECM components (COII, aggrecan) in cultured AIA chondrocytes.

## Methods

### Experimental induction of AIA in rats and evaluation

Male Sprague–Dawley rats (three months old, 140–160 g) were obtained from Experimental Animal Center of Anhui Medical University. The rats were housed in plastic cages under standardized conditions of temperature and humidity. After 7-day acclimatization, the rats were randomly divided into sham control and AIA experimental group (n = 15). Complete Freund’s adjuvant (CFA) was prepared by suspending heat-killed *Mycobacterium butyricium* (Detroit Laboratory, MI, USA) in sterile paraffin oil (10 mg/mL). Rats in AIA group received a single intradermal injection of 0.1 mL CFA into the right hind paw [14,15]. The equal volume of saline was given to rats in sham group. The injection day was regarded as day 0. The volume of non-injected (left) hind paw was measured by a plethysmograph apparatus on day 0 (basic value) and day 10, 14, 18, 22, 26 respectively. The secondary paw swelling was defined as volume changes on day 0 and each time point (ΔmL). All protocols were approved by Ethic Committee and Animal Experimental Committee of Anhui Medical University.

### Tissue preparation

Animals were euthanized on day 26 after AIA induction. The left knee joints were removed, trimmed, fixed in 4% paraformaldehyde and then decalcified in 10% EDTA for 2 weeks. The tissues were embedded in paraffin and sliced (5 μm) for the histological examination and immunohistochemical analysis. Fresh cartilage tissues quickly obtained from right knee joints were used to perform real-time PCR and isolate articular chondrocytes.

### Histological examination of articular cartilage damage

Paraffin sections of knee joints were stained with hematoxylin and eosin (HE) and evaluated by two trained observers unknown the sample source. The severity of cartilage damage to the femoral condyle and tibial plateau was semi-quantified with Osteoarthritis Research Society International (OARSI) scores [16,17], which is defined as assessment of combined disease grade (0–6 points) and disease stage (0–4 points), with a combined assessment of disease severity and extent (0–24 points). The average score of three specimens from each knee joint was used for statistical analysis. Safranin O staining of knee joints sections was also performed to assay proteoglycans expression in articular cartilage.

### Immunohistochemical analysis of Hh signal related genes (Shh, Ptch1, Smo and Gli1)

The procedures of immunohistochemistry were performed with classical protocols [18]. Briefly, tissue sections were hydrated, rinsed and microwave-treated in citrate-buffered saline. Sections were treated with 3% hydrogen peroxide followed by incubated in 5% goat serum. Subsequently, sections were incubated overnight at 4°C with primary antibodies (Santa Cruz, CA, USA) against Shh, Ptch1, Smo and Gli1 (1: 100). Sections were incubated with biotinylated IgG (1:200) and avidin-biotin horseradish peroxidase complex (1:200) at 37°C for 30 min. Sections were visualized with diaminobenzidine, dehydrated, hyalinized in xylene and mounted with neutral gum. Staining scores were calculated by semi-quantitative optical analysis. Cell membranes, cytoplasm, and/or nuclei that contained yellow or brown granules were considered positively stained cells. Five fields per slide were evaluated the number of positive cells and total cells, and the positive expression rates were calculated. The positive expression rate (%) was defined as (number of positive cells/total cells) × 100%. The proportion of positive cells was graded as follows: negative expression (−), < 5% positive cells; weakly positive (+), 5-25% positive cells; moderately positive (++), 25-75% positive cells; strongly positive (+++), > 75% positive cells [19].

### RNA isolation and real-time quantitative PCR (Q-PCR)

Total RNA was extracted from cartilage tissues using Trizol method according to the protocol (Invitrogen, CA, USA). cDNA were synthesized by a RevertAid First Strand cDNA Synthesis Kit (Thermo Scientific, PA, USA). Q-PCR was performed by SYBR Green PCR Kit (Applied Biosystems, USA) and an ABI Prism 7000 Sequence Detector system in 25 μl volume for 40 cycles: 15 s at 95°C; 60 s at 64°C (Shh, Smo, COII) or 60°C (Ptch1, Gli1, aggrecan). The primer sequences for target genes were synthesized by Sangon Biotech Company (Shanghai, China) and listed in Table 1. The 2^-ΔΔCt^ method was used to determine relative amount of mRNA [20], and the result from each sample was normalized against that of β-actin. The relative amplification efficiencies of the primers were tested and shown to be similar.

**Table 1:** **Primer sequences used in the study**

**Gene**	**5′-3′ primer sequence**	**Product size (bp)**	**GenBank accession**
Shh	Forward: TCCGATGTGTTCCGTTACC	100	NM_017221.1
	Reverse: AACCTTGCCTGCTGTTGC		
Ptch1	Forward: CACCAAGTGATTGTGGAAGC	102	NM_053566.1
	Reverse: CTGTTGCCGAGAGTTCAAGG		
Smo	Forward: ATGCGTGTTTCTTTGTGGGC	133	NM_012807.1
	Reverse: ACACAGGATAGGGTCTCGCT		
Gli1	Forward: AACTCCACGAGCACACAGG	106	NM_001191910.1
	Reverse: GGCAGTCCGTCTCATACACA		
COII	Forward: TCAAGTCGCTGAACAACCAG	116	NM_012929.1
	Reverse: GTCTCCGCTCTTCCACTCTG		
aggrecan	Forward: GCAGCACAGACACTTCAGGA	137	NM_022190.1
	Reverse: CCCACTTTCTACAGGCAAGC		
β-actin	Forward: TTGCTGACAGGATGCAGAA	101	NM_031144.3
	Reverse: ACCAATCCACACAGAGTACTT		

### Isolation of articular chondrocytes and identification

Articular chondrocytes were isolated and prepared by the method of trypsin and collagenase digestion as described previously [21,22]. Briefly, small minced cartilage from knee joint (about 1 mm^3^) was digested with 0.25% trypsin for 30 min and with 0.2% type II collagenase for 3 h in cell incubator, respectively. Then the isolated cells were pipetted through 200-mesh nylon mesh into a sterile centrifuge tube. After wash with PBS, the freshly isolated chondrocytes were suspended in DMEM supplemented with 10% fetal calf serum (FBS) and incubated in a flat bottomed culture bottle at 37°C, 5% CO_2_ for 5 days. Adherent cells were trypsinized, split and recultured in medium. The chondrocytes of passages 1–3 were used in our studies. The cultured chondrocytes were identified by immunocytochemical stain of COII and toluidine blue staining of glycosaminoglycan. The chondrocytes were fixed with 4% paraformaldehyde and permeated with 0.5% Triton X-100. The detailed procedure of immunocytochemical stain of COII was similar to the immunohistochemical steps described above. Some chondrocytes were incubated with 1% toluidine blue dye solution at 37°C for 30 min, washed subsequently with distilled water, 95% ethanol and xylol, and mounted.

### Shh, Ptch1, Smo, Gli1, COII and aggrecan mRNA levels in cultured chondrocytes

The cultured cells were divided into different groups including chondrocytes from sham rats (sham chondrocytes), chondrocytes from AIA rats (AIA chondrocytes) and AIA chondrocytes with cyclopamine treatment (AIA chondrocytes + cyclopamine-10 μM). Cyclopamine (LC Laboratories, USA) was dissolved at 20 g/L in ethanol and diluted to the final concentration using DMEM with 10% FBS. Cultured articular chondrocytes were seeded in 6-well plates at a density of 2 × 10^8^ cell/L and incubated at 37°C, 5% CO_2_ for 24 h growth, then the culture medium was aspirated and the cells were treated with 1 mL vehicle or cyclopamine (10 μM) for another 48 h. The total RNA was extracted from the cultured articular chondrocytes. cDNA was synthesized and used for PCR. The levels of Shh, Ptch1, Smo, Gli1, COII and aggrecan mRNA were detected by real-time PCR as mentioned above.

### Statistical analysis

Statistical analysis was performed by SPSS software. Values are presented as mean ± SEM. The data were analyzed by Independent-Samples T test or One-Way analysis of variance followed by LSD post hoc test. Correlation between mRNA levels of Shh, Ptch1, Smo, Gli1 in cartilage and the severity of cartilage damage of AIA rats (indicated by OARSI scores, COII and aggrecan mRNA levels) were determined by Pearson’s correlation test. *p* < 0.05 was considered to be statistically significant.

## Results

### Evaluation of AIA in rats

**Figure 1:**
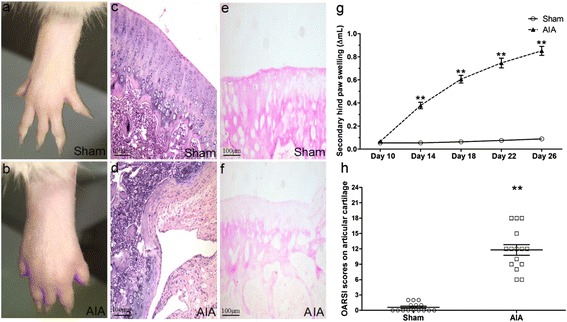
**Evaluation of AIA in rats. (a, b)** Representative photos of non-injected hind paw on day 26 after arthritis induction. **(c, d)** Representative histopathologic photos of knee joints sections with HE staining (×100). **(e, f)** Representative histopathologic photos of knee joints sections with Safranin O staining (×100). **(g)** The changes of secondary hind paw swelling on different time point (days 10, 14, 18, 22, 26). **(h)** OARSI scores on articular cartilage of knee joints. Data are mean ± SEM (n = 15). ^**^ *p* < 0.01 compared with the sham group.

Typical photos of non-injected hind paw from sham and AIA rats were taken on day 26 after AIA induction (Figure 1a,b). There was a significant increase of secondary hind paw swelling (i.e. secondary inflammation) on different time points (days 14, 18, 22, 26) (Figure 1g). Photomicrographs of knee joint paraffin sections with HE staining illustrated the severity of cartilage damage in AIA rats. No cartilage destruction was seen in the knee joints from sham rats (Figure 1c). On the contrary, typical pathological characteristics of cartilage damage including cartilage loss, articular cartilage zone thinness and articular surface roughness were apparently found in AIA rats (Figure 1d). Safranin O staining further revealed that proteoglycans were positively expressed in articular cartilage of the sham rats (Figure 1e) while were faintly observed in the AIA rats (Figure 1f). In addition, OARSI scores on articular cartilage in AIA group were obviously higher than those in sham group, indicating the articular cartilage damage of AIA rats (Figure 1h).

### Shh, Ptch1, Smo and Gli1 proteins were highly expressed in cartilage of AIA rats

**Figure 2:**
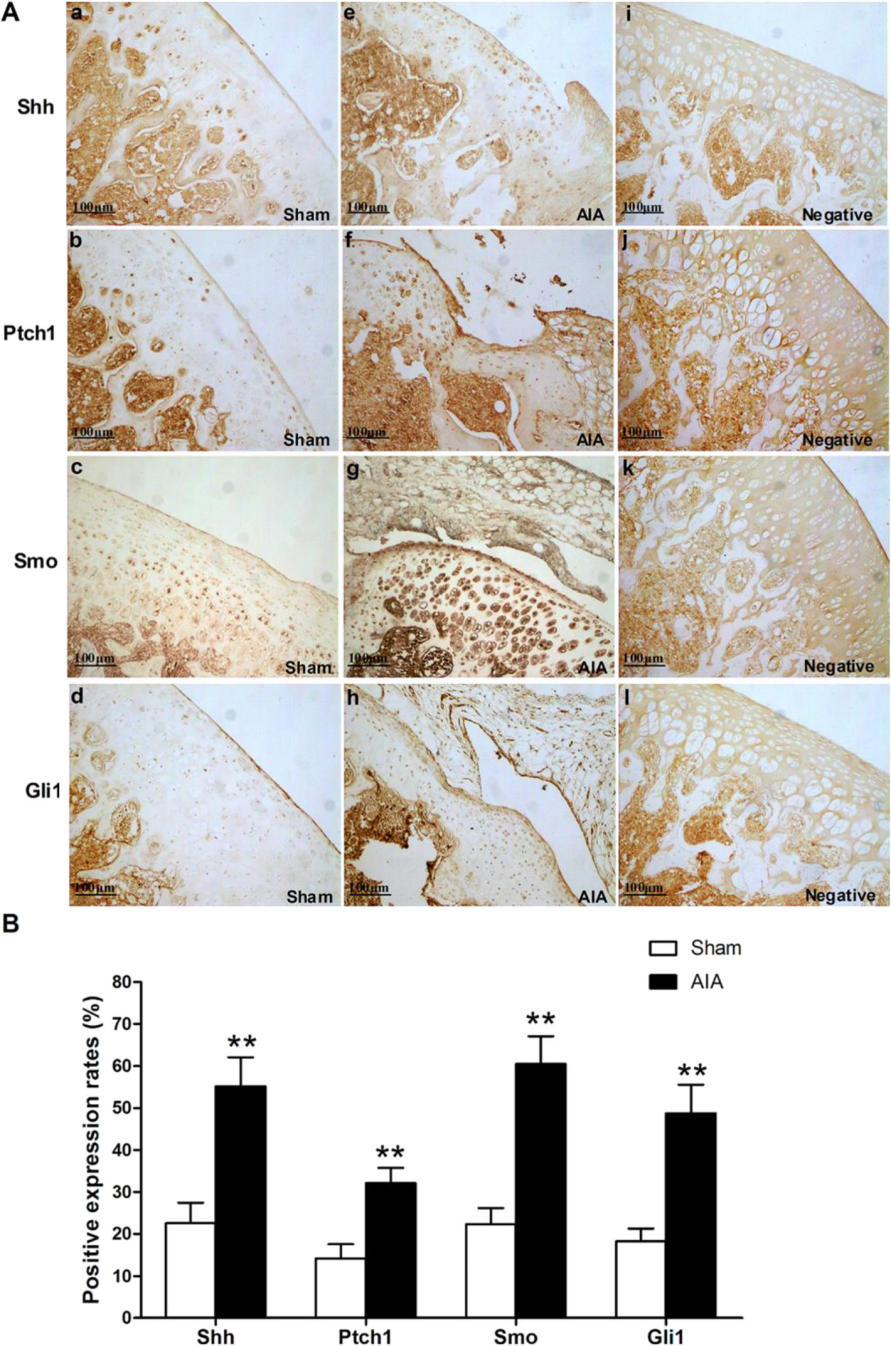
**Immunohistochemistry analyses for Shh, Ptch1, Smo and Gli1 in articular cartilage of knee joints. (A)** Typical images of Shh, Ptch1, Smo and Gli1 expression in articular cartilage from sham and AIA rats (×100). Shh **(a)**, Ptch1 **(b)**, Smo **(c)** and Gli1 **(d)** were expressed at low levels in articular cartilage from the sham rats, while relatively strong positive staining of Shh **(e)**, Ptch1 **(f)**, Smo **(g)** and Gli1 **(h)** were seen in the AIA rats. Negative controls were enclosed to show the antibody specificity **(i, j, k and l)**. **(B)** The positive expression rates of Shh, Ptch1, Smo and Gli1 in articular cartilage from sham and AIA rats. The positive expression rate is defined as (number of positive cells/total cells) × 100%. Data are mean ± SEM (n = 15). ^**^ *p* < 0.01 compared with the sham group.

We performed immunohistochemical assay to study the localization and relative protein levels of Shh, Ptch1, Smo and Gli1 in articular cartilage of sham and AIA rats. Typical photos of immunohistochemistry were respectively shown in Figure 2A. Shh staining was primarily localized at the cell membrane and cytoplasm of chondrocyte (Figure 2A-a,e). Ptch1 and Smo were mainly expressed in the cytoplasm of chondrocyte (Figure 2A-b,f and Figure 2A-c,g). Gli1 staining was mostly observed in the nucleus of chondrocyte (Figure 2A-d,h). Shh, Ptch1, Smo and Gli1 showed similar expression patterns in the cartilage of sham rats and AIA rats. However, more positive stained chondrocytes of the above proteins were seen in the cartilage of AIA rats (Figure 2A-e,f,g and h). Negative controls were also enclosed to show the antibody specificity (Figure 2A-i,j,k and l). We performed a statistical analysis on the positive expression rates of interest proteins in cartilage from the sham and AIA group. Our results showed that the positive expression rates of Shh, Ptch1, Smo and Gli1 in AIA rats were all significantly increased compared with those in sham rats (Figure 2B).

The results of semi-quantitative analyses of Shh, Ptch1, Smo and Gli1 expressions in cartilage of the sham and AIA rats were shown in Table 2. Our results indicated that the rate of moderately (++) and strongly (+++) positive expression of Shh, Ptch1, Smo and Gli1 (80%, 53.3%, 86.7% and 73.3% respectively) in AIA rats was higher than that in the sham rats (33.3%, 20%, 40% and 20% respectively). However, no strongly (+++) positive expression of Ptch1 was found in the sham and AIA group.

**Table 2:** **The semi-quantitative analysis results of Shh, Ptch1, Smo and Gli1 protein expression in articular cartilage**

**Group**	**Shh**				**Ptch1**				**Smo**				**Gli1**			
	**−**	**+**	**++**	**+++**	**−**	**+**	**++**	**+++**	**−**	**+**	**++**	**+++**	**−**	**+**	**++**	**+++**
Sham	2	8	5	0	2	10	3	0	2	7	6	0	1	11	3	0
AIA	0	3	6	6	0	7	8	0	0	2	8	5	0	4	6	5

Detected by immunohistochemical assay.

### Changes of Shh, Ptch1, Smo, Gli1, COII and aggrecan mRNA expressions in articular cartilage of AIA rats

**Figure 3:**
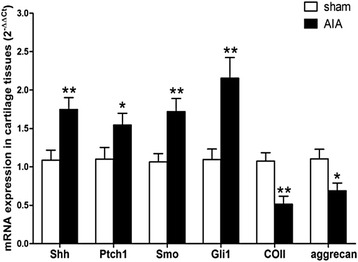
**The mRNA levels of Shh, Ptch1, Smo, Gli1, COII and aggrecan in articular cartilage from sham and AIA rats.** Detected by real-time Q-PCR. Data are mean ± SEM (n = 15). ^*^ *p* < 0.05, ^**^ *p* < 0.01 compared with the sham group.

The mRNA expression of Hh pathway related genes (Shh, Ptch1, Smo and Gli1) and ECM components (COII and aggrecan) in articular cartilage was measured by real time Q-PCR and analyzed statistically. The mRNA levels of Shh, Ptch1, Smo and Gli1 were significantly higher in articular cartilage of the AIA rats than those of the sham rats (Figure 3). The Q-PCR results of Shh, Ptch1, Smo and Gli1 mRNA were consistent with the results of the protein staining in immunohistochemistry. In addition, compared with the sham group, COII and aggrecan mRNA levels were significantly lower in articular cartilage in AIA group (Figure 3).

### Relationships between Shh, Ptch1, Smo, Gli1 mRNA levels in cartilage and the severity of cartilage damage in AIA rats

As shown in Table 3, mRNA levels of Hh signal related genes in articular cartilage of AIA rats significantly correlated positively with OARSI scores (Shh, *r* = 0.561, *p* = 0.029; Gli1, *r* = 0.565, *p* = 0.028), and significantly correlated negatively with COII mRNA level (Shh, *r* = −0.680, *p* = 0.005; Ptch1, *r* = −0.572, *p* = 0.026; Smo, *r* = −0.533, *p* = 0.033; Gli1, *r* = −0.587, *p* = 0.021) and aggrecan mRNA level (Shh, *r* = −0.576, *p* = 0.025; Ptch1, *r* = −0.590, *p* = 0.021; Gli1, *r* = −0.765, *p* = 0.001). Weak correlations including Ptch1 and Smo with OARSI scores, Smo with aggrecan mRNA levels were also found without reaching statistical significance. Our results imply that the increased mRNA levels of Shh, Ptch1, Smo and Gli1 in cartilage might be associated with the severity of cartilage damage of AIA rats.

**Table 3:** **Correlations between mRNA levels of Shh, Ptch1, Smo and Gli1 in articular cartilage and the severity of cartilage damage in AIA rats (n = 15), indicated by OARSI scores on articular cartilage, COII and aggrecan mRNA levels in articular cartilage**

**mRNA levels**		**Shh**	**Ptch1**	**Smo**	**Gli1**
OARSI scores	*r*	0.561 (*)	0.346	0.489	0.565(*)
	*p*	0.029	0.207	0.064	0.028
COII mRNA levels	*r*	−0.680 (**)	−0.572 (*)	−0.533 (*)	−0.587 (*)
	*p*	0.005	0.026	0.033	0.021
Aggrecan mRNA levels	*r*	−0.576 (*)	−0.590 (**)	−0.353	−0.765 (**)
	*p*	0.025	0.021	0.197	0.001

The correlation analysis was performed by the Pearson’s correlation test. Asterisks (*) show the significance of the correlation (two tails). ^*^ *p* < 0.05; ^**^ *p* < 0.01.

### Effects of cyclopamine on mRNA levels of Hh signal, COII and aggrecan in AIA articular chondrocytes *in vitro*

**Figure 4:**
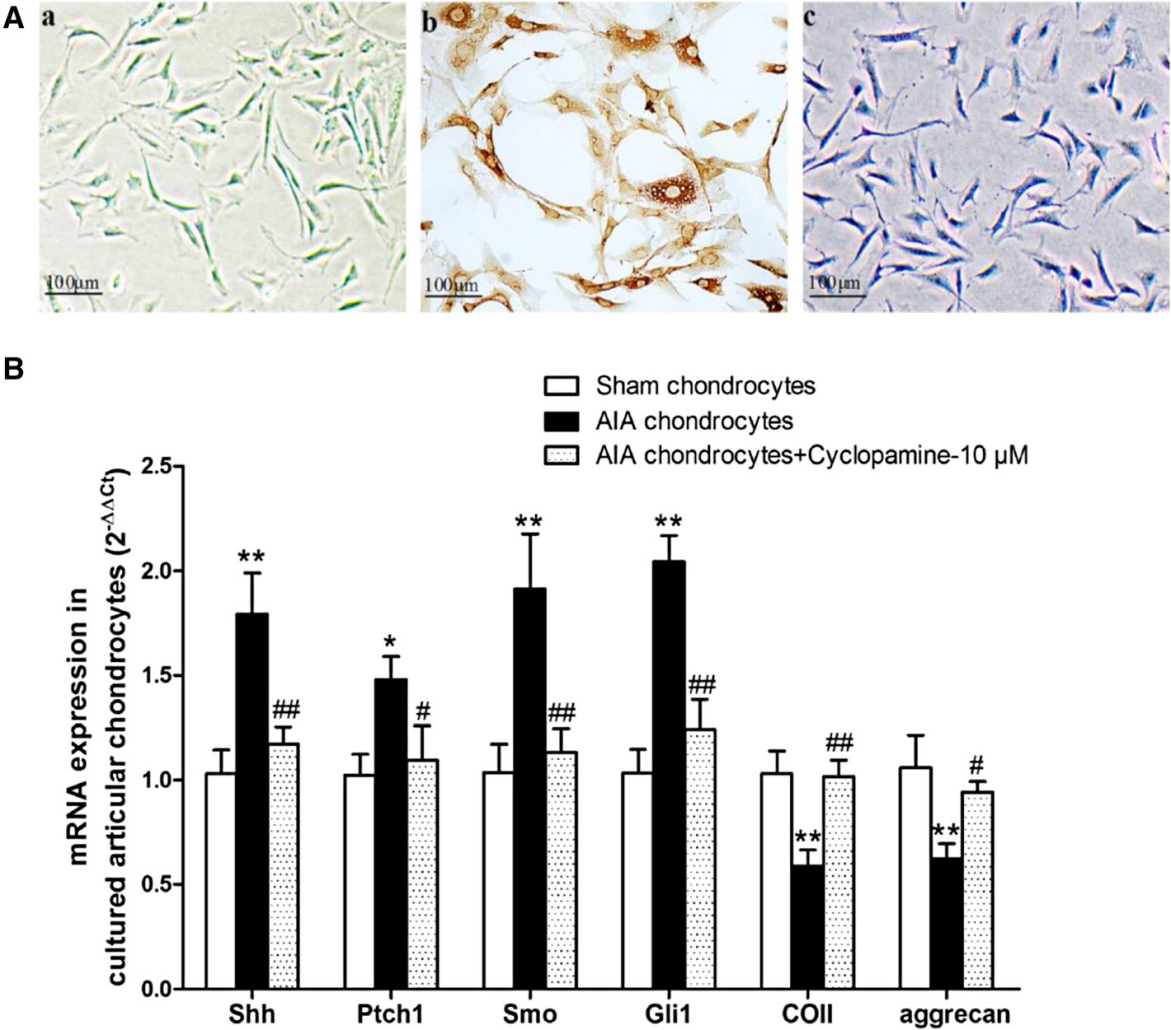
**Effects of cyclopamine on mRNA levels of Hh signal, COII and aggrecan in AIA articular chondrocytes** ***in vitro*** **. (A)** Identification of cultured articular chondrocytes. **(a)** Morphology of chondrocytes after one passage (×100). **(b)** Positive immunocytochemical staining of COII (×100); **(c)** Positive toluidine blue staining of glycosaminoglycan (×100). **(B)** Cyclopamine (10 μM) decreased Shh, Ptch1, Smo and Gli1 mRNA levels, while increased COII and aggrecan mRNA levels in cultured AIA articular chondrocytes, detected by real-time Q-PCR. Sham and AIA chondrocytes were alone cultured in DMEM added 10% FBS. Data are mean ± SEM (n = 6). ^*^ *p* < 0.05, ^**^ *p* < 0.01 compared with sham chondrocytes. ^#^ *p* < 0.05, ^##^ *p* < 0.01 compared with AIA chondrocytes.

The morphology of cultured chondrocytes after one passage displayed a long spindle shape (Figure 4A-a). The cultured cells were identified to be chondrocytes by positive immunocytochemical staining of COII (Figure 4A-b) and positive toluidine blue staining of glycosaminoglycan (Figure 4A-c). Consistent with our findings in cartilage tissues *in vivo*, increased Shh, Ptch1, Smo and Gli1 mRNA levels and decreased ECM components (COII, aggrecan) mRNA levels were observed in AIA chondrocytes compared with those in sham chondrocytes (Figure 4B). Cyclopamine (10 μM) treatment significantly reversed the abovementioned changes of interest gene expressions in AIA chondrocytes (Figure 4B), indicating that effective inhibition of Hh signal might directly promote ECM production of AIA chondrocytes *in vitro*.

## Discussion

In this paper, we investigated the expression of Hh signal in cartilage and its pathological role in cartilage damage of AIA rats. Our results revealed that Shh, Ptch1, Smo and Gli1 showed higher mRNA and protein levels in cartilage of AIA rats than those of sham rats. The elevated mRNA levels of Hh signal were associated with the severity of cartilage damage in AIA rats. *In vitro*, cyclopamine effectively inhibited the activation of Hh signal and increased the gene expression of COII and aggrecan in cultured AIA chondrocytes.

AIA model shares many pathological features with RA including inflammation, synovial hyperplasia and aggressive pannus formation which lead to cartilage and bone damage [23]. AIA model is used extensively to elucidate the pathogenic mechanisms relevant to RA and to identify potential targets for therapeutic intervention [24,25]. Therefore, rat AIA is the suitable experimental model for the present study to investigate the possible pathological role of Hh signal in cartilage damage of RA. Herein, we successfully established AIA rat model, as evidenced by increased secondary paw swelling, histopathological changes of knee joint and elevated OARSI scores on cartilage.

Chondrocytes as the unique cellular component in cartilage is important to control ECM synthesis and degradation. Chondrocytes injury may be of pathogenetic significance in arthritis development including RA and OA [26]. Hh signal regulates chondrocyte proliferation and differentiation, finally influences the synthesis of collagen and aggrecan in ECM [27,28]. Some studies using knockout mice revealed that activation of Hh pathway caused a remarkable decrease of cartilage thickness and proteoglycans content, while Hh inhibition increased cartilage thickness and proteoglycans content [29,30]. Over-activated Hh signal in chondrocytes promoted chondrocyte hypertrophy and cartilage damage in OA [11,12]. In the current study, immunohistochemistry results showed that Hh signal related proteins (Shh, Ptch1, Smo and Gli1) were expressed in a similar pattern in articular cartilage of sham and AIA rats (i.e. Shh at cell membrane and cytoplasm, Ptch1 and Smo at cytoplasm, and Gli1 at nucleus). However, these proteins were highly expressed in articular cartilage of AIA rats, while their expression levels were relatively lower in sham rats. Real-time PCR results revealed that the mRNA levels of Shh, Ptch1, Smo and Gli1 genes were significantly elevated in cartilage of AIA rats than those of sham rats. In addition, correlation analyses were performed to elucidate whether the increased mRNA levels of Hh signal related genes were related to the severity of cartilage damage of AIA rats. Our results indicated that Shh and Gli1 correlated positively with OARSI scores, Shh, Ptch1, Smo and Gli1 correlated negatively with COII mRNA levels, and Shh, Ptch1 and Gli1 correlated negatively with aggrecan mRNA levels. The above results suggested that Hh signal was over-activated in the articular cartilage of AIA rats and might play an important role in cartilage damage.

Cyclopamine is a steroidal alkaloid obtained from separation and extraction of the corn lily (*Veratrum californicum*). As a classical specific inhibitor of Hh pathway by directly binding to Smo, it has been extensively used *in vivo* and *in vitro* experiments [31]. In the present study, we successfully isolated and prepared articular chondrocytes which were identified by positively stain of COII and glycosaminoglycan. Then we investigated the possible effect of cyclopamine on mRNA expression of Hh signal related genes and ECM components (COII and aggrecan) in cultured AIA articular chondrocytes. Consistent with our findings in cartilage tissues *in vivo*, the mRNA levels of Shh, Ptch1, Smo and Gli1 were significantly elevated, while the mRNA levels of COII and aggrecan were remarkably reduced in cultured AIA chondrocytes than those in sham chondrocytes. Cyclopamine treatment significantly suppressed the activation of Hh signal and increased COII, aggrecan mRNA expressions in cultured AIA chondrocytes. It is well known that COII is the main structural collagen in cartilage and aggrecan is the major structural proteoglycans in articular cartilage ECM [32]. Our results suggested that inhibition of Hh signal by cyclopamine might directly promote ECM production of AIA chondrocytes *in vitro*.

## Conclusions

Taken together, our study revealed that Hh signal pathway was over-activated in the articular cartilage of AIA rats, and the up-regulated Hh signal was apparently associated with the severity of cartilage damage. *In vitro*, inhibition of Hh signal by cyclopamine could promote COII and aggrecan mRNA expressions in cultured AIA chondrocytes. The present study suggests, for the first time, that Hh signal pathway underlies the pathogenesis of cartilage destruction in RA. Further research is needed to investigate the potential therapeutical effect of cyclopamine and its molecular mechanisms on AIA rats.

## Abbreviations

AIA: Adjuvant-induced arthritis
CFA: Complete Freund’s adjuvant
COII: Type-II collagen
ECM: Extracellular matrix
FBS: Fetal calf serum
FLS: Fibroblast-like synoviocytes
Gli1: Glioma-associated oncogene homolog 1
Hh: Hedgehog
OA: Osteoarthritis
Ptch1: Patched homologue 1
RA: Rheumatoid arthritis
Shh: Sonic hedgehog
Smo: Smoothened
